# “ECT should never stop”: Exploring the experiences and recommendations of ECT clinical directors and anesthetists about ECT during the COVID-19 pandemic

**DOI:** 10.3389/fpsyt.2022.946748

**Published:** 2022-09-14

**Authors:** Grace Branjerdporn, Shanthi Sarma, Laura McCosker, Vanessa Dong, Donel Martin, Colleen Loo

**Affiliations:** ^1^Mental Health and Specialist Services, Gold Coast Hospital and Health Service, Southport, QLD, Australia; ^2^Medicine Department, Faculty of Health Sciences and Medicine, Bond University, Robina, QLD, Australia; ^3^School of Medicine and Dentistry, Griffith University, Brisbane, QLD, Australia; ^4^Discipline of Psychiatry and Mental Health, School of Clinical Medicine, University of New South Wales, Sydney, NSW, Australia; ^5^Black Dog Institute, Sydney, NSW, Australia

**Keywords:** electroconvulsive therapy, ECT, COVID-19, pandemic, experiences, recommendations, survey, anesthesia

## Abstract

Electroconvulsive therapy (ECT) is an effective treatment option for severe, treatment-resistant, and life-threating psychiatric illness. The COVID-19 pandemic has significantly disrupted ECT services. Services across North America, Europe, and Australia have reported decreased ECT delivery, and changes in the ways ECT is delivered. This study aimed to identify the impacts of COVID-19 on ECT services globally by exploring clinicians' experiences about ECT during the pandemic, and their recommendations for reducing the negative impacts of the pandemic. Data were collected using an electronic, cross-sectional survey, which included elicitation of free-text responses. The survey was open from March to November 2021. Clinical directors in ECT services, their delegates, and anesthetists were invited to participate. This paper reports the qualitative analysis of responses provided. Fifty-two participants provided qualitative response/s; 74.5% were clinical directors or their delegates, and 25.5% were anesthetists. Greater than one-third of participants were from Australia/New Zealand, and there was also representation from North America, Europe, and the UK. Participants' responses were detailed, averaging 43 words. Three themes were identified: (1) *Service provision*, about the importance of ECT services continuing during the pandemic, (2) *Preparedness*, through guidelines and environmental design, and (3) *Personal protection*, about strategies to increase staff safety. This is the first multi-site, international study to document the experiences and recommendations of ECT clinical directors and anesthetists about the effect of COVID-19 on ECT practice. The findings inform evidence-based practice, and ensure people with major psychiatric illnesses continue to receive ECT during the pandemic.

## Introduction

Electroconvulsive therapy (ECT) is an effective treatment option for severe, treatment-resistant, and life-threating psychiatric illnesses including depression, schizophrenia, and bipolar disorder (1). The necessity of ECT in the treatment of these illnesses was supported by professional bodies worldwide (1–3). As with most health services, and especially procedure-based services, the COVID-19 pandemic has significantly disrupted ECT services, with services across North America, Asia, Europe, and Australia reporting decreases in ECT delivery and major changes in how ECT is delivered (4–6). This qualitative study explored the experiences of ECT clinical directors and anesthetists, globally, about ECT during the pandemic, and its impact on clinical services.

Several causal factors have been identified which were associated with decreased ECT during the pandemic. Many services classified ECT as an “elective procedure” early in the pandemic, to control viral spread and conserve resources (7). Staff redeployment, sickness, and quarantine/isolation mandates led to staffing shortages at many services (4, 5). ECT processes such as electrode placement and anesthesia require direct clinician-patient contact (8), and the need for new infection control measures also had major impacts. Staff spent considerable time donning/doffing personal protective equipment (PPE) (4, 5), and—at least at the beginning of the pandemic**—**PPE shortages disrupted service provision (8). ECT treatment rooms required extra ventilation and cleaning between patients (5). ECT anesthesia aerosol-generating procedure (9), and this limited where ECT could be performed and necessitated changes in anesthetic technique (10, 11). In many services, time-intensive pre-treatment COVID-19 screening was introduced (11). ECT treatment intervals were extended, ECT prioritized for the most severely ill patients, and ECT delivery canceled, with negative impacts on clinical outcomes (4–6). Anecdotal reports and single service studies, multi-site national studies (4, 7), and bi-national studies (5, 6), have been published about the impact of these factors on ECT provision. However, large multi-national and qualitative data is lacking.

Understanding the impacts of the COVID-19 pandemic on ECT delivery is vital to informing evidence-based practice for planning for future pandemics. This study qualitatively surveyed the experiences of ECT clinical directors and anesthetists, around the world, about the provision of ECT during the pandemic. It also captures their recommendations for reducing the impacts of the pandemic.

## Methodology

### Participants and procedure

The authors assert that all procedures contributing to this work comply with the ethical standards of the relevant national and institutional committees on human experimentation, and with the Helsinki Declaration of 1975, as revised in 2008. All procedures involving human subjects/patients were approved by the Gold Coast Health Human Research Ethics Committee (HREC/2020/QGC/70077).

This qualitative study took a descriptive phenomenological approach, exploring participants' lived experiences with a phenomena—in this case, ECT service provision during the pandemic (12). Data were collected using an electronic, cross-sectional survey, which was open from March 2021 to November 2021. The survey was administered in Microsoft Office Forms. It included multiple-choice (quantitative) and free-text (qualitative) questions; this paper reports on the qualitative data, with the quantitative data to be reported elsewhere. A participant information sheet was displayed on the first page of the survey. Completion of the survey implied consent to participate in the study. No identifiable data was collected.

Clinical service directors (i.e., clinical leads of ECT services who are likely to be consultant psychiatrists), their delegates, and ECT anesthetists were invited to participate in the survey. Additional eligibility criteria were that participants were adults (i.e., over 18 years of age), and had sufficient comprehension of the English language to complete the survey. A link to the survey was distributed to ECT-related mailing lists and forums including the Clinical Alliance and Research in Electroconvulsive Therapy (CARE) Network (13), the Royal Australian and New Zealand College of Psychiatry Section for ECT and Neurostimulation, the International ECT Network, the ECT Accreditation Service (ECTAS), and state ECT Committees. A snowballing approach to recruitment was adopted whereby recipients were encouraged to distribute the survey link to their networks. Sample size was determined based on sufficient sample size required for the quantitative analyses, which are reported elsewhere.

The survey contained questions about: (a) the participant and the service they worked in, and (b) the impacts of the COVID-19 pandemic on their service, including in relation to ECT delivery, access to ECT, access to anesthetic medications, staffing, PPE, clinical impacts, and participants' recommendations. There were forty-one multiple-choice (quantitative) questions. At the end of the survey, there were also two free-text (qualitative) questions:

1. *Would you have done things differently or have any recommendations for future pandemics?*
2. *Any other comments?*

This paper reports findings from the qualitative data obtained from these questions.

### Analysis

Quantitative data about participants and their services were analyzed descriptively in Stata, version 20. Percentages were calculated using the “number of possible positive responses” as the denominator. Qualitative data were analyzed thematically, using the framework developed by Braun and Clarke (14). Qualitative data analysis was undertaken manually by [insert author's initials]. Data from the two questions were pooled, then coded. These codes were inductively analyzed, then grouped into broader themes and sub-themes. Inter-rater checks took place until a consensus on themes and representative quotes was achieved. In this paper, quotes are reported verbatim, with only minor edits to ensure confidentiality. The average response length of the qualitative responses (number of words) was also calculated. Responses such as “no”, “none”, and “N/A” were excluded from the word count.

## Results

### Participants

Table 1 provides demographic information about the participants and their ECT services. Of the 112 participants who completed the survey, 52 participants provided response/s to the qualitative questions analyzed in this study. Of these, 74.5% (*n* = 38) were ECT clinical directors or their delegates, and 25.5% (*n* = 13) were anesthetists involved in ECT.

**Table 1:** Demographic information about the study participants and their ECT services.

**Demographic variable**	***N***	**%**
**Participants' role in ECT service delivery (n** **=** **51)**		
Clinical director	38	74.5%
Anesthetist	13	25.5%
**Type of ECT service (*****n*** **=** **51)**		
Public	36	70.6%
Private	13	25.5%
Other	2	3.9%
**Location of ECT service—country (*****n*** **=** **52)**		
Australia	20	38.5%
North America	12	23.1%
Europe	8	15.4%
United Kingdom	8	15.4%
Central/South America	2	3.8%
Asia	1	1.9%
Africa	1	1.9%
**Location of ECT service—region (*****n*** **=** **52)**		
Metropolitan	36	69.2%
Regional	15	28.8%
Rural/remote	1	1.9%
**Location of ECT service—COVID-19 community transmission (*****n*** **=** **51)**		
Yes, community transmission	4	7.8%
No, community transmission	47	92.2%
**Location of ECT service—COVID-19 hotspot status (*****n*** **=** **51)**		
Low-risk hotspot	9	17.6%
Moderate-risk hotspot	4	7.8
High-risk hotspot	38	74.5%
**Location of ECT service—COVID-19 lockdown/s (*****n*** **=** **50)**		
Yes, the service has experienced lockdown/s	45	90.0%
No, the service has not experienced lockdown/s	5	10.0%

The majority of participants (70.6%, *n* = 36) were based in public hospitals. Many (38.5%, *n* = 20) were from Australia and New Zealand, and there was also high representation from North America (23.1%, *n* = 12), Europe (15.4%, *n* = 8), and the United Kingdom (UK) (15.4%, *n* = 8). There were few participants (<10%, *n* = 4) from South/Central America, Asia, or Africa. The majority of participants (69.2%, *n* = 36) were from a service located in a metropolitan area. Most participants (92.2%, *n* = 4 7) worked in regions with community transmission of COVID-19, and most (82.3%, *n* = 32) worked in regions which were considered “medium risk” or “high risk” COVID-19 hot spots. Most participants (90.0%, *n* = 45) worked in regions which had experienced at least one lockdown at some point prior to the administration of the survey.

### Word count analysis and nature of responses

Pooled, the average length of the free-text response was 43 words (SD: 44 words, range: 3–190 words). Most participants provided response/s that were considered and insightful, with evidence of strong beliefs indicated by tone and structure (e.g., capital letters, exclamation marks).

### Themes

From the qualitative responses provided by survey participants, three key themes were identified: (1) *Service provision*, (2) *Preparedness*, and (3) *Personal protection*:

#### Theme 1—Service provision

This theme explored *service provision* in relation to participants' views about the importance of ECT remaining available during the pandemic, their attempts to find solutions and advocate for service continuation, and problems they identified with service discontinuation:

##### Subtheme 1.1—Importance of ECT services continuing

Many participants (*n* = 25) provided strongly-worded responses arguing that ECT services must continue during the pandemic. One participant said: “*We should avoid shutting down ECT services in future pandemics and raise awareness about ECT as an essential procedure”* (P56). Others commented:

- “*Healthcare managers and providers should be sensitized to the fact that ECT is an essential treatment” (P47)*.
- “*Make sure it is known on an international organization level that ECT is a life-saving procedure and should not be considered elective”* (P78).
- “*ECT is essential and life-saving. It should NEVER be stopped”* (P63).

##### Subtheme 1.2—Solutions

Study participants reported that they and their services had sought solutions to ensure ECT treatment continued during the pandemic. One participant explained: “*One patient was reluctant to come in for maintenance treatment and relapsed as a result. We utilized another service which kept up with ECT throughout during our brief hiatus to ensure that patients who required ECT got it”* (P103). Another participant proposed using a: “*triage rating scale e.g., Edmonton Triage Scale—Pandemic Version”* (P42) to prioritize patients most in need of ECT services, where services were limited. A third participant suggested: “*Continue ECT as an essential procedure for critically ill patients* [only]. *Adjust the procedure and technique to the new situation. Don't cease ECT”* (P49).

##### Subtheme 1.3—Advocacy

Participants reported that ECT staff acted assertively, advocated with service managers, and worked collaboratively to ensure the continuation of ECT where appropriate. Often, efforts were successful. One participant said: “*when an anesthesiologist went to infection control to stop ECT I made my case to the medical director … with a risk/benefit for not continuing ECT and willingness to make changes as needed for safety. ECTs were only halted for 2 days after we worked out our plans”* (P40). Another participant commented: “*We were able to convince hospital executives that ECT is not an elective procedure—once this occurred, ECT practice returned to normal”* (P81). When reflecting on the challenges of advocacy, one participant said: “*Be okay with and prepared for things to change quickly. Remember to take slow deep breaths, smile, be kind and forgiving”* (P40).

##### Subtheme 1.4—ECT discontinuation

Many of the participants worked in services where ECT treatment was discontinued, either partially (*n* = 13, 25.0%) or fully (*n* = 17, 32.7%). Participants frequently reflected on the reasons for this. One said: “*The ECT service was quieter during the pandemic period. Hospitals were not allowed visitors, patients had to be screened prior to coming into hospital, patients had to wear masks while in hospital and patient leave was restricted. These restrictions dissuaded people who were not acutely unwell to forego ECT treatment”* (P64). The quantitative data also revealed multiple other complex causes for service discontinuation, which will be reported elsewhere.

Participants in services that discontinued ECT treatment often expressed their perception that the decision was ill-advised. One participant commented: “*The suspension of activity with ECT was one month and a half; we have learned a lot and we are prepared not to suspend ECT in future pandemics”* (P58). Another participant reflected: “*With hindsight, anesthetists and ODPs (operating department practitioners* = *anesthetic assistants) should not have all been withdrawn from semi-urgent surgical lists and ECT lists to man ICUs* [intensive care units]*, because our ICUs were never swamped in the manner feared. Patients missed out on … ECT because of this miscalculation, whilst anesthetists twiddled their thumbs on overstaffed, half-full ICUs. This must never happen again”* (P102).

Participants also reflected on their belief that decisions about the discontinuation of ECT services were made by administrators, rather than ECT clinicians. One participant said: “*Many non-psychiatric clinicians and administrators lack understanding of the utility and necessity of ECT and this results in stigma and frustration toward the ECT psychiatrists and our patients”* (P78). In a later response, this participant commented: “*We need more physicians and psychiatrists making administrative decisions and less administrators”* (P78).

Participants were acutely aware that the discontinuation of ECT services could lead to negative clinical outcomes for patients. One participant commented: “*We learnt as we went with the guiding principle of ECT being an essential treatment. We were aware that for some of our outpatient/maintenance patients relapse could lead to admission which had its own risks (our unit had 3 COVID outbreaks)”* (P103). Participants also recognized that lack of access to ECT services could lead to patient deaths, and some participants worked in services that experienced deaths. Again, this is explained in greater depth by the quantitative data.

#### Theme 2—Preparedness

The theme of *Preparedness* relates to participants' views on the importance of guidelines to support clinical decision-making during the pandemic, valuing the team's perspective in the development of these guidelines, and considering the design of ECT treatment settings:

##### Subtheme 2.1—Guidelines to support clinical decision-making

The most common feedback received from participants (*n* = 30) was that they desired guidelines from peak bodies about ECT in pandemic conditions. One participant commented: “*I think that there needs to be a central national co-ordinated response, similar to the CDC* [Centers for Disease Control and Prevention]…* There needs to be a central authority directing the response”* (P88). This participant also commented on inconsistency in existing guidelines: “*the rules around COVID patients varied throughout the day, all of which simply heightened healthcare worker's fears”* (P88). Another said: “*Next time at least some evidence of management involvement or some national guidance* [in decision-making] *would be nice”* (P19).

Participants also recommended that ECT experts, rather than non-clinical managers, drive guideline development. One participant explained: “*Strong advocacy and role by ECT experts is important in advising* [our] *approach during COVID”* (P14). Another commented on specific professions which may be involved: “*Standardized PPE and COVID procedural protocols should be discussed and drafted by* [a peak body] *and then implemented nation-wide. Other medical specialities are not well informed enough or have the technical and scientific know-how. Anesthetists should spearhead any form of COVID protocol”* (P94).

Participants felt that guidelines would have saved time in having to advocate with service managers for the continuation of ECT services. One participant explained: “*So much time was wasted trying to develop plans and negotiate with executives rather providing appropriate clinical care. Having a national or state-wide guidance would have provided reassurance and saved time”* (P22). Participants also believed that guidelines would have supported the preparation of services for operating in pandemic conditions. One participant commented: “*By the time* [our city] *had some minor community transmission, and some restrictions, we had... practiced our procedures and processes and were more confident, primarily because we had a lot more knowledge than at the start”* (P65). Further, participants felt that guidelines would have alleviated burden for each service to devise its own strategy or approach. One participant said: “*It would have been helpful if the state health service had a plan re*[garding] *ECT service during COVID times, and that way we would know our place and how to prepare, instead of all services having to prepare as if they were it!”* (P10).

##### Subtheme 2.2–Valuing the team's perspective

While having guidelines was important, many participants highlighted that balancing this with the individual service context and clinical reasoning was also prudent. One participant explained: “*Each situation is unique and the clinical team need to make decisions on the information they have available at the time. Our decisions were rational and logical and even in retrospect I would not change the approach we took”* (P64). Another participant commented: “*Team communication and frequent reassessment of the situation and procedures is paramount”* (P99).

##### Subtheme 2.3—Collaboration

Working effectively across disciplines was perceived as important: “*Collaborative working with our team and the support of the microbiology department, public health specialists and the perioperative directorate was key to ensuring the safe delivery of care”* (P103). Another participant emphasized the need to: “*facilitate clear communication of policies between different services”* (P57), for consistency.

##### Subtheme 2.4—ECT treatment settings

Participants suggested a range of environmental considerations for the design and flow of ECT treatment settings, to reduce the airborne transmission of COVID-19 and help to prepare for ECT delivery in pandemic conditions. Participants described the importance of “*increase*[ing] *ventilation in the treatment rooms”* (P91) and ensuring that there was “*less crowding in waiting areas”* (P35). Participants also suggested: “*increas*[ing] *the number of rooms available to perform ECT”* (P91), and: “*design*[ing] *ECT theaters to make them meet the standards* [for] *aerosolising procedures”* (P12). Further changes were suggested to patient flow. One participant explained: “*In my service it was necessary to interrupt the ECT activity due to the location of the ECT area in the acute hospitalization ward and the entry of the COVID-19 into the ward. Having an ECT unit located outside the ward would have made things easier”* (P56). The approaches services took to making ECT treatment settings COVID-safe are detailed in the quantitative data.

#### Theme 3—PPE and protection

In the third theme, participants outlined their views about the importance of PPE and fit-testing, COVID-19 testing, and vaccination in enabling the continuation of ECT services:

##### Subtheme 3.1—PPE and fit-testing

Participants frequently reinforced that having timely access to adequate PPE was imperative for staff delivering ECT services. Participants commented:

- “*More PPE earlier!!!”* (P21).
- “*Adequate PPE is the key to uninterrupted ECT services”* (P34).

Participants felt that hospital and health services—particularly, mental health staff—were slow with their uptake of fit testing. One participant said: “*Unfortunately our health care networks were very slow to realize the relevance and organize fit testing for staff. At this stage I believe most mental health workers in our facility have not been fit tested”* (P90). Another participant further explained this problem: “*Fit checking and fit testing came very late in the pandemic…* [There were] *issues around PPE and not being able to do fit testing as we had a limited supply of masks (and gowns) and therefore couldn't ‘waste' them on testing”* (P88). Participants felt that guidelines about PPE were useful.

##### Subtheme 3.2—COVID-19 testing

Many participants voiced their belief that testing patients prior to ECT was helpful and reassuring for staff. One participant commented: “*I wish our service had continued with pre-treatment COVID swab and isolation as a requirement for patients having ECT* **– ***it made everyone feels safer to know the patient had tested negative”* (P84). However, other participants observed that COVID-19 testing was not undertaken consistently, or even at all, in some ECT services. One participant reflected: “*I know COVID was rife in our psychiatric inpatient wards with one ward having a 100% infection rate at one point… We still don't PCR* [polymerase chain reaction test] *all our patients before each treatment and we still have very high infection rates… i.e., 35,000 new cases per day”* (P115).

##### Subtheme 3.3—Vaccination

At the time the survey was delivered, most regions were beginning COVID-19 vaccination campaigns for health workers and general populations. Many participants found this a reassuring and positive step. One participant commented: “*since the early part of the year* [2021] *almost all our staff have been double vaccinated and the majority of our patients”* (P115). Another participant reflected: “*Vaccination has been a game changer and everyone is much more relaxed now”* (P57).

## Discussion

This is the first multi-site, international study to document the qualitative experiences and recommendations of ECT clinical directors and anesthetists about ECT during the COVID-19 pandemic. Study data were collected between March 2021 and November 2021, and by this time ECT services had been operating pandemic conditions for at least 1 year. Participants were able to provide detailed, insightful responses to the qualitative questions. It is noteworthy that the findings in this paper were developed from data collected using just two free-text (qualitative) questions at the end of a survey. For this reason, specific qualitative analysis was deemed important and necessary. Despite major differences in location, culture, and health system structure, all participants discussed broadly similar ideas.

The participants were steadfast in their belief that ECT services should continue during the pandemic, and this position was supported by professional bodies globally (15–19). For people with severe, treatment-resistant, and life-threating psychiatric illness, ECT is medically necessary; without it, reductions in patients' health-related quality of life, clinical deterioration (resulting in hospitalization and rehospitalisation), and avoidable mortality are common (6, 20). Considering the pandemic caused an overall worsening of pre-existing psychiatric illness as well as a significant increases in first-onset psychiatric illnesses (21, 22), disruptions to ECT services came at a time when these services were needed the most.

The participants told how they staunchly advocated for ECT services to continue during the pandemic, and developed a variety of solutions to enable them to do so. Basic actions such as referring patients to other sites when ECT services closed, triaging patients by clinical need when services were limited, and otherwise advocating that services remain operational are relevant to all ECT services. Decisions about discontinuing ECT services are often driven by lack of understanding of the role of ECT as a treatment, and stigma (20). ECT staff have a responsibility to provide expert knowledge to ensure such decisions are fully-informed.

The participants felt that ECT clinicians had a limited role in decision-making about ECT service delivery. These comments came from services worldwide, but primarily in Australia/New Zealand. This is in contrast to another multi-national study, which found that in two-thirds of the ECT services surveyed clinicians fulfilled key decision-making roles (6). ECT services will see a backlog of need as they adjust to COVID-normal operations, and waves of infection will continue to disrupt service delivery (5). Considering clinicians' expert perspectives is vital to ensuring ECT services respond appropriately to these challenges—and, indeed, that these services remain operational throughout. This study highlights that clinicians' frontline experience in managing ECT services during the pandemic has given them considerable insight into how to do this safely and effectively—including in relation to the design of ECT treatment settings, the use of PPE, COVID-19 testing, and vaccination. Clinicians should be involved in decision making in these matters.

Although local perspectives and experience are important, this study shows that ECT staff also value higher-level guidance about the delivery of ECT services in pandemic conditions. It must be noted that a number of professional bodies have published guidelines for the delivery of ECT during the COVID-19 pandemic (15–19). However, participants expressed frustration that many of these came too late and some lacked input from ECT clinicians. This frustration is, perhaps, evidenced by the large volume of publications from the first year of the pandemic discussing ECT services' own local approaches to service delivery (8, 11, 23–26). While local approaches, relevant to specific contexts, are important, they are often *ad hoc* and arbitrary, and in the longer-term may increase infection risk and cause considerable stress to staff (4). Contributing to the process of documenting learnings into higher-level guidance—specifically, evidence-based practice guidelines—is important for ECT services in all regions. In ECT services in Singapore, for example, highly effective responses to COVID-19 pandemic in 2020 were directly informed by previous responses to the Severe Acute Respiratory Syndrome (SARS) outbreak in 2003, captured in national guidelines (8).

Evidence based guidelines may also help to avoid the unnecessary discontinuation of ECT services in future pandemics. Unnecessary service discontinuation—often, due to inaccurate surge planning for COVID-19 urgent/critical/intensive care settings, and the subsequent excessive redeployment of ECT staff—was an interesting issue raised in this study. It is also an issue highlighted in the broader literature—for example: in the early months of the pandemic in the UK and Ireland, around half of the ECT services surveyed had essential staff redeployed to COVID-19 care settings which were expected to reach critical capacity, but which never did so (5). It is, of course, simplistic to criticize such decisions with the benefit of hindsight, but evidence-based guidelines could help to ensure such errors do not occur again.

The findings of this study must be understood in the context of its methodological limitations. The survey was administered in English, and excluded ECT staff without sufficient comprehension of the language in its written form. It is possible that these staff may have had different experiences and recommendations. Because it is unknown if and how participants distributed the survey link through their networks, it is not possible to calculate a response rate. It must also be acknowledged that this study excludes the voices of consumers—that is, people who use ECT services—although these are vital to informing evidence-based practice. Two members of the research team were ECT clinical directors and, although they were reflexive throughout the study, their experience may have influenced their interpretations.

This is the first multi-site, international study to document the qualitative experiences and recommendations of ECT clinical directors and anesthetists about ECT during the COVID-19 pandemic. Participants supported the continued provision of ECT services during the pandemic. Through the themes of *Service provision, Preparedness*, and *Personal protection*, participants offered insights into how ECT can be delivered in a safe and effective way despite the limits created by pandemic conditions. These findings are vital to ensuring that people with severe, treatment-resistant, and life-threating psychiatric illnesses continue to receive the care they need, as ECT services adjust to COVID-normal operation.

## Data availability statement

The raw data supporting the conclusions of this article will be made available by the authors, without undue reservation.

## Ethics statement

The studies involving human participants were reviewed and approved by Gold Coast Health Human Research Ethics Committee. Completion and submission of the online survey implied consent from participant's that the data could be used for the purposes of this research project, as per national legislation and the institutional requirements.

## Author contributions

SS and GB developed the research question. GB, SS, VD, DM, and CL designed, piloted, and implemented the data collection survey. GB, SS, and LM completed the data analysis. All authors contributed to the manuscript draft and approved the final manuscript.

## Conflict of interest

The authors declare that the research was conducted in the absence of any commercial or financial relationships that could be construed as a potential conflict of interest.

## Publisher's note

All claims expressed in this article are solely those of the authors and do not necessarily represent those of their affiliated organizations, or those of the publisher, the editors and the reviewers. Any product that may be evaluated in this article, or claim that may be made by its manufacturer, is not guaranteed or endorsed by the publisher.

## References

[1] Royal College of Psychiatrists. Statement on Electroconvulsive Therapy. Royal College of Psychiatrists. (2017). Available online at: https://www.rcpsych.ac.uk/docs/default-source/about-us/who-we-are/electroconvulsive-therapy—ect-ctee-statement-feb17.pdf?sfvrsn=2f4a94f9_2 (accessed May 01, 2022).

[2] Royal Australian New Zealand College of Psychiatrists (RANZCP). Electroconvulsive Therapy (ECT). RANZCP. (2019). Available online at https://www.ranzcp.org/news-policy/policy-and-advocacy/position-statements/electroconvulsive-therapy-(ect) (accessed May 01, 2022).

[3] American Psychiatric Association (APA). What is Electroconvulsive Therapy (ECT)? APA. (2019). Available online at: https://www.psychiatry.org/patients-families/ect (accessed May 02, 2022).

[4] Demchenko I Blumberger D Flint A Anderson M Daskalakis Z Foley K. Electroconvulsive therapy in Canada during the first wave of COVID-19: results of the 'what happened' national survey. J ECT. (2022) 183:52–9. 10.1097/YCT.0000000000000801 34519681 PMC8875437

[5] Braithwaite R Chaplin R Sivasanker V. Effects of the COVID-19 pandemic on provision of electroconvulsive therapy. BJPsych Bulletin. (2021) 46:1–4. 10.1192/bjb.2021.43 33977894 PMC9344553

[6] Kwan E Le B Loo CK Dong V Tor PC Davidson D. The impact of COVID-19 on electroconvulsive therapy: a multisite, retrospective study from the clinical alliance and research in electroconvulsive therapy and related treatments network. J ECT. (2022) 38:45–51. 10.1097/YCT.0000000000000800 34387286 PMC8875438

[7] Maixner DF Weiner R Reti IM Hermida AP Husain MM Larsen D. Electroconvulsive therapy is an essential procedure. Am J Psychiatry. (2021) 178:381–2. 10.1176/appi.ajp.2020.20111647 33979536

[8] Tor PC Ahh P Koh Dsh YM. ECT in a time of COVID-19. J ECT. (2020) 28:527–9. 10.1177/1039856220953705 32924540

[9] Purushothaman S Fung D Reinders J Garrett-Walcott S Buda M Moudgil V. Electroconvulsive therapy, personal protective equipment and aerosol generating procedures: A review to guide practice during Coronavirus Disease 2019 (COVID-19) pandemic. Australas Psychiatry. (2020) 28:632–5. 10.1177/1039856220953699 32910692

[10] Jagadheesan K Walker F Danivas V Itrat Q Lakra V. COVID-19 and ECT - A Victorian perspective. Australas Psychiatry. (2021) 29:540–5. 10.1177/10398562211014224 33993747

[11] Ramakrishnan VS Kim YK Yung W Mayur P. ECT in the time of the COVID-19 pandemic *Australas Psychiatry*. (2020) 28:527–9. 32924540 10.1177/1039856220953705

[12] Liamputtong P. Qualitative Research Methods. 5th ed. Docklands: Oxford University Press. (2020).

[13] Martin DM Galvez V Lauf S Dong V Baily SA Cardoner N. The clinical alliance and research in electroconvulsive therapy network: an Australian initiative for improving service delivery of electroconvulsive therapy. J ECT. (2018) 41:7–13. 10.1097/YCT.0000000000000435 28658011

[14] Braun V Clarke V. Using thematic analysis in psychology. Qual Res Psychol. (2006) 3:77–101. 10.1191/1478088706qp063oa

[15] Royal Australian New Zealand College of Psychiatrists (RANZCP). Information About the Provision of ECT Treatment During the COVID-19 Pandemic. RANZCP. (2020). Available online at: https://www.ranzcp.org/files/resources/college_statements/practice_guidelines/ranzcp-information-ect-and-covid.aspx. (accessed May 03, 2022).

[16] Royal College of Psychiatrists. Continuing to provide ECT during the COVID-19 pandemic. Continuing to Provide ECT During the COVID-19 Pandemic. Royal College of Psychiatrists. (2020). Available online at: https://www.rcpsych.ac.uk/docs/default-source/about-us/covid-19/ect-covid-19-briefing-may-20-submitted.pdf?sfvrsn=dd39d278_2 (accessed May 05, 2022).

[17] International Society for ECT Neurostimulation (ISEN). Position Statement on ECT as an Essential Procedure During COVID-19. ISEN, 2020. Available online at: https://www.isen-ect.org/covid-19-essential-procedure (accessed May 06, 2022).

[18] Royal College of Anaesthetists (RCOA). Anaesthesia for ECT During the COVID-19 Pandemic. RCOA. (2020). Available online at: https://icmanaesthesiacovid-19.org/anaesthesia-for-ect-during-the-covid-19-pandemic (accessed May 07, 2022).

[19] American Psychiatric Association (APA). Electroconvulsive Therapy as an Essential Procedure. APA. (2020). Available online at: https://www.psychiatry.org/File%20Library/Psychiatrists/APA-Guidance-ECT-COVID-19.pdf (accessed May 04, 2022).

[20] Demas ML. Electroconvulsive therapy and triaging during reduced access and the COVID-19 pandemic: a personal perspective. J ECT. (2020) 36:226–8. 10.1097/YCT.0000000000000713 32618743

[21] Phiri P Ramakrishnan R Rathod S Elliot K Thayanadan T Sandle N. An evaluation of the mental health impact of SARS-CoV-2 on patients, general public and healthcare professionals: a systematic review and meta-analysis. Lancet. (2021) 31:806. 10.1016/j.eclinm.2021.100806 33842872 PMC8022621

[22] Xiong J Lipsitz O Nasri F Liu LMW Gill H Phan L. Impact of COVID-19 pandemic on mental health in the general population: a systematic review. J Affect Disord. (2020) 277:55–64. 10.1016/j.jad.2020.08.001 32799105 PMC7413844

[23] Bryson EO Aloysi AS. A strategy for management of ECT patients during the COVID-19 pandemic. J ECT. (2020) 36:149–51. 10.1097/YCT.0000000000000702 32826544 PMC7299101

[24] Colbert SA McCarron S Ryan G DMM. Images in clinical ECT: Immdiate impact of COVID-19 on ECT practice. J ECT. (2020) 36:688. 10.1097/YCT.0000000000000688 32433120 PMC7188035

[25] Surve RM Sinha P Baliga SP Radhakrishnan M Karan N Anju JL. Electroconvulsive therapy services during COVID-19 pandemic. Asian J Psych. (2021) 59:e102653. 10.1016/j.ajp.2021.102653 33845300 PMC8022516

[26] Grover S Sinha P Sahoo S Arumugham S Baliga S Chakrabarti S. Electroconvulsive therapy during the COVID-19 pandemic. Indian J Psychiatry. (2020) 62:582–4. 10.4103/psychiatry.IndianJPsychiatry_335_20 33678842 PMC7909042

